# A health-based long term vision to face air pollution and climate change

**DOI:** 10.3389/fpubh.2022.947971

**Published:** 2022-08-24

**Authors:** Gudrun Weinmayr, Francesco Forastiere

**Affiliations:** ^1^Institute of Epidemiology and Medical Biometry, Ulm University, Ulm, Germany; ^2^Environmental Research Group, School of Public Health, Imperial College, London, United Kingdom; ^3^IFT-National Research Council (CNR), Palermo, Italy

**Keywords:** air pollution, climate change, prevention, systems thinking, coproduction, climate change mitigation

## Introduction

Our society needs to rapidly speed up its transition to sustainable energy production and use. This is more urgent than ever in the present war period with an international crisis of energy supply. Many scientists are concerned that the current policy actions are insufficient to prevent global temperature from rising more than 1.5 or 2 degrees when consequences would be unpredictable and partly irreversible.

Science has already provided strong evidence that a radical change is urgently needed.

The research behind the updated worldwide WHO Air Quality Guidelines (1) and the IPCC Sixth Assessment Report (AR6) (2, 3) is overwhelming. More than two decades of scientific investigations have demonstrated that exposure to air pollutants has a huge impact on population health, not only on respiratory and cardiovascular diseases but also on reproductive health and chronic conditions like dementia and diabetes. Air pollution was the 4^th^ leading risk factor for early death worldwide in 2019, surpassed only by high blood pressure, tobacco use, and poor diet (4). Similarly, non-optimal temperatures, one of the many changes induced by climate warming, are associated with a substantial mortality burden worldwide (5). Climate change and related events are also an ever-increasing threat to mental health and thus coping abilities in our changing world (6).

Here we point out the window of opportunity for a swift energy transition in the present crisis leading to a sustainable way of living on our planet. We suggest several concepts rooted in systems thinking^1^ and systems approach to population health as enablers for this transition.

## Immediate changes in energy policies: Phase-out of fossil fuels, promotion of renewable sources, reduction of energy needs

Overall the message of the AR6 on mitigation is clear: “we need to act now, not tomorrow but today” (8). The present energy crisis is, paradoxically, a great opportunity. There are immediate actions that help to respond to the energy crisis but, at the same time, are useful to face climate and air pollution challenges.

The radical reduction of fossil fuel use in energy systems should now be expedited giving utmost priority to a connected grid of (decentralized) renewable energy production from sustainable technologies with proven efficiency (solar, wind, geothermal, hydropower). Immediate positive effects on air pollution reduction, and therefore health, will counteract the annual premature 4.14 million deaths, attributable to ambient air pollution worldwide (4). Note that this estimate may be considered an underestimation of the true impact of fossil fuel related air pollution given the non-linearity of the exposure-response function with steeper slope at lower concentrations and slower drop-off in slope at higher concentrations (9). The International Energy Agency (IEA) already formulated proposals to reduce fossil fuel dependence (https://www.iea.org/reports/a-10-point-plan-to-reduce-the-european-unions-reliance-on-russian-natural-gas). As the IPCC report indicates, this transition to low-carbon energy provides immediate economic opportunities for the investors (profit) and business (decreased costs).

Furthermore, reduction of energy needs is mandatory. It could be achieved by adjusting indoor temperatures (lower in the cold and higher in the warm seasons), with targeted subsidies for insulation of buildings, and electrification of transport–in the public and private sector–alongside a reduction in the number of motorized vehicles. Again, the IEA has released recommendations for advanced economies to cut oil demand by 2.7 million barrels a day within a few months (https://www.iea.org/reports/a-10-point-plan-to-cut-oil-use). The simple and easy to understand recommendations for the mobility sector include a drastic reduction of speed limits, of transport in general by increasing work from home, and of travel by airplanes.

## A long-term vision

A successful, sustainable energy transition toward zero-emission needs systemic prerequisites, with regulations (air pollution limits, strict building regulations, limitation of the numbers and weight of circulating vehicles, radical shift from subsidies of fossil fuels to renewables etc.) and a reorganization of infrastructure (for renewable sources of energy, heating, electric mobility, cycling…). The involvement of civic society, a key player in consumption and saving patterns, is mandatory for designing successful policies (10).

This long-term perspective covers virtually all sectors: industry; transport; buildings; urban systems; agriculture; forestry and other land use; and energy systems. The respective mitigation options encompass the change in consumption/production patterns and technological shifts. Examples of the former are the shift to active transport in cities, energy savings in the building sectors and a circular economy–more efficient recovery and recycling of mineral and other resources used in all sectors are needed.

Most options have potential large health co-benefits, and they, even alone, might be strong drivers of change. For example, co-benefits of interventions regarding urban planning, transport, and diet have been suggested for Italy (11), a country where PM2.5 pollution accounts for 50,856 premature deaths per year (8.6% of total deaths) and the contribution of heat to mortality was 14,521 in 2015 (2.3% of total deaths) (12). Urban planning offers great potential for disease prevention and climate change mitigation by preventing the heat island phenomena with an increase in green spaces absorbing atmospheric CO_2_ and reducing the demand for energy for cooling. Green spaces are also associated with higher levels of physical activity, reduced obesity and improved mental health. Promoting active transport through the provision of safer cycle lanes and pedestrian routes has the multiple effects of mitigating greenhouse gas emissions, preventing diseases related to air pollution, and increasing physical activity, which, in turn, is related to various health benefits including mental health. Agriculture contributes a large share of total greenhouse gas emissions mainly related to animal breeding and methane production by ruminants. Non-fossil methane is a 80-fold stronger greenhouse gas than CO_2_ when projected over the next 20years due to its higher radiative efficiency combined with its shorter lifetime of 12 years (13) (vs. more than 100s−1,000 years for CO_2_) and will be amenable to rapid short term mitigation. However, the shorter half-life also implies that its impact declines over time (only 27 fold stronger than CO_2_ over 100 years) and therefore has to be accompanied by long term strategies for long-lived greenhouse gases (CO_2_, N_2_O and others). In addition, manure spreading is a source of ammonia, which is transformed into PM_2.5_, a toxic air pollutant. Finally, high consumption of red meat is related to chronic degenerative diseases, particularly cardiovascular diseases. A strong reduction in meat consumption would thus be related to strong benefits for health and the planet. Good examples of how cities should consider the multiple societal, environmental, and health benefits of municipal actions to reduce GHG emissions are in the experience of the C40 cities network, where local actions across 25 global cities prevent a total of 2,655 premature deaths and 9,275 annual hospitalizations, reduce asthma cases, increase physical activity, and improve wellbeing across all cities (14). The methodology for estimating the health impact of climate policies is improving (15) and a systematic analysis of the co-benefit of the US decarbonization policy is available (16).

The great challenge, requiring a rethinking of our economic activities, is to implement the measures needed in an equitable way that benefits human (and planetary) health. The causes for climate change and existing inequities are largely the same and tackling them will probably bring the most benefits for a healthy global humanity (17).

To achieve such a transition, a holistic approach is necessary, having health at its heart, involving different disciplines, sectors and stakeholders including civic society.

## Systems-thinking and co-production

People live in complex social and economic systems. Policy needs to reflect this dynamic complexity (18) and thus consider interactions between sectors, feedback loops, synergies and trade-offs, potential unanticipated side-effects, and, importantly, advert lock-ins that block favorable developments for years or even decades to come (19). Examples of the two latter are fiscally favored diesel fuel to mitigate greenhouse gases, which led to widespread adoption of diesel cars in Europe and related emissions that are detrimental to health, and the development of car-friendly rather than inhabitant-friendly cities. Inhabitant-friendly cities would prioritize the health, social cohesion and quality of life of their inhabitants rather than accessibility by car (20).

As an example of systems thinking, Berry et al. (7) developed a comprehensive conceptual framework regarding the different physical impacts of climate change on mental health taking into account distal, intermediate and proximate harms mediated by various factors in the domains of personal and public resources, social dynamics and community functioning, business conduct and governance. To encompass all these complexities, transdisciplinary co-production^2^ is vital, combining interdisciplinary science and professional collaboration with the knowledge of the local community sectors (stakeholders and citizens), including the most vulnerable which are often primarily affected by status-quo and intervention. In recent time, more examples and studies of how to put co-production can be set into practice have become available (21–24).

Importantly, interventions need to be accompanied by monitoring e.g., with carefully chosen indicators or other assessment tools (24–26), to follow up on desired endpoints but also to keep an eye open for inadvertent side events. Health needs to be at the center of these considerations which could follow the ideas of the recently proposed Planetary Health Watch (27).

To address root causes, strategies should not only have a technological but also a social innovation component. New technologies need resources (e.g., minerals, rare earth elements): these are not endless and heavy reliance on them may create new dependencies and market instabilities; extraction and production practices may have considerable negative impacts on the health of workers and populations on site. Furthermore, rebound effects inducing increased consumption of new technologies often thwart energy savings.

Equally important are therefore sufficiency^3^ and resource-economic measures. For example, electric vehicles are one puzzle piece, only, among several others such as active and public transport, reorganizing mobility as service rather than product ownership.

For policy to be effective, it should be evidence-based. In light of the above, this evidence base needs to take account of systems complexities and dynamics, and therefore may need to be created/refined alongside the implementation and take account of local context in a co-production approach. This will aid the science and the achievement of equitable outcomes and acceptability by civic society, an important factor for political will that so often is perceived as lacking. On the other side, political will that comes without necessary evidence or a testing phase accompanied by scientific evaluation and coproduction can cause large damages as in the example of the sudden ban of importation of pesticides and synthetic fertilizers in Sri Lanka (28).

Importantly, for coproduction to be effective devoted personnel/funds, interdisciplinary training and exchange, transparency and good communication building trust are essential (24, 25). Also to be reconsidered are current systems of research funding and career development which are mostly based on competitive principles rather than fostering cooperation for participatory science. Notwithstanding these challenges, (Public) Health professionals should play an essential role in transdisciplinary coproduction.

## Role of individual behavior and systemic approaches

The transition will not work without social consent, and the role of individuals, in their decisions on how to behave and consume, is much discussed. Professionals in (public) health know well this debate from the individual vs. systems approach for prevention. Smoking cessation is a prominent example showing the limits of individual capacities and the resistance of vested interests to contextual policy interventions.

The challenge we face for the transition toward sustainability is even greater with manifold vested interests and required changes touching upon many, seemingly natural habits of everyday life and deeply rooted, partly subconscious, values. Solutions need to facilitate sustainable behavior and make them become the new “normal.” Enabling these new modes of action will strengthen the transition because an individual's actions are the strongest determinants of behavior, more so than rational decisions and emotions (29): individuals acting sustainably are more likely to believe in the importance of counteracting the threats of climate change and to act correspondingly, than individuals whose lifestyles counteract sustainability. This could lead to strengthening feedback loops. If more and more individuals are adopting sustainable behavior and activities, eventually a social tipping point^4^ could be reached leading to necessary rapid disruptive system changes.

However, to achieve this it seems essential to make everyday choices for sustainability easy and convenient. The political toolbox can consist of regulations such as the European directive on the energy efficiency of household appliances, taxes (however to be designed to enhance equity), corresponding infrastructure (e.g., more bike lanes instead of ever more motor vehicle lanes) and nudging^5^ initiatives, e.g., by making the sustainable option the default. All these initiatives aim to make sustainable behaviors the new routine, i.e., “ecoroutine” (30). Good and inclusive communication, viable alternatives and adequate time-frames (without losing ambition) may be considered key elements for success, and sometimes even bold regulation may buy in acceptability with time as was the case with safety belt regulations that seem so natural today. Good communication can focus on achievable short-term benefits, notably the health co-benefits of climate mitigation: reduction of air pollution will immediately improve health and individual climate action is an effective remedy for climate anxiety, which affects already parts of the population and will likely become even more prevalent over time as the climate warms further. Current concerns in society such as increased perception of climate warming in the current summer, or of energy availability linked to the Ukraine war can be valuable allies in mobilizing society e.g., for dietary changes and energy savings.

## Conclusion

The national health systems and individual health professionals, both in public health and in clinical medicine, have a highly relevant role, which has been extensively recognized. Prevention services and physicians could be leading the changes to establish a well-structured prevention dialogue with citizens and patients that include constant and motivating recommendations for everyday healthy mobility, energy use, and nutritional choices. In addition, they should raise their voice as a group of highly trusted professionals to advocate for related structural changes, enabling the “ecoroutine,” which, facilitated by systemic conditions and reinforced by health professionals, will be a key driver of the change.

## Author contributions

GW and FF conceived the idea of the manuscript, researched literature, and wrote the manuscript. All authors contributed to the article and approved the submitted version.

## Conflict of interest

The authors declare that the research was conducted in the absence of any commercial or financial relationships that could be construed as a potential conflict of interest. The reviewer PV declared a shared parent affiliation with the author FF to the handling editor at the time of the review.

## Publisher's note

All claims expressed in this article are solely those of the authors and do not necessarily represent those of their affiliated organizations, or those of the publisher, the editors and the reviewers. Any product that may be evaluated in this article, or claim that may be made by its manufacturer, is not guaranteed or endorsed by the publisher.

## Footnotes

^1^Systems thinking has been defined as “a set of ‘synergistic analytic skills’ used to help describe a complex set of interacting factors that produce outcomes, to predict their behavior and to formulate interventions to achieve desired (and avoid pernicious) results” (7).

^2^Co production has been defined as “an asset-based approach to public services that enables people providing and people receiving services to share power and responsibility, and to work together in equal, reciprocal and caring relationships. It creates opportunities for people to access support when they need it, and to contribute to social change.” https://copronet.wales/.

^3^The IPCC in its 6^th^ AR distinguishes sufficiency from efficiency as follows: “Sufficiency is about long-term actions driven by non-technological solutions, which consume less energy in absolute terms. Efficiency, in contrast is about continuous short-term marginal technological improvements.”

^4^Points “within a social-ecological systems (SES) at which a small quantitative change inevitably triggers a non-linear change in the social component of the SES, driven by self-reinforcing positive-feedback mechanisms, that inevitably and often irreversibly lead to a qualitatively different state of the social system” (31).

^5^Definition according to Thaler and Sunstein (32): “A nudge, as we will use the term, is any aspect of the choice architecture that alters people's behavior in a predictable way without forbidding any options or significantly changing their economic incentives. To count as a mere nudge, the intervention must be easy and cheap to avoid. Nudges are not mandates. Putting fruit at eye level counts as a nudge. Banning junk food does not.”

## References

[1] WHO. WHO Global Air Quality Guidelines: Particulate Matter (?PM2.5 and PM10)?, Ozone, Nitrogen Dioxide, Sulfur Dioxide and Carbon Monoxide. 34662007

[2] IPCC. Climate Change 2022: Impacts, adaptation, and vulnerability. In: Portner H-O Roberts DC Tignor M Ploczanska ES Mintenbeck A Alegria A Craig M Langsdorf S Loschkr S Moller V Okem A Rama B, editors. Contribution of Working Group II to the Sixth Assessment Report of the Intergovernmental Panel on Climate Change. Cambridge University Press (2022). Available online at: https://www.ipcc.ch/report/ar6/wg2/downloads/report/IPCC_AR6_WGII_FullReport.pdf (accessed August 12, 2022).

[3] IPCC. Climate change 2022: Mitigation of climate change. In: Shukla PR Skea J Slade R Alkhourdajie A van Diemen R McCollum D Pathak M Some S Vyas P Fradera R Belkacemi Hasija A Lisboa G Luz S Malley J, editors. Contribution of Working Group III to the Sixth Assessment Report of the Intergovernmental Panel on Climate Change. Cambridge; New York, NY: Cambridge University Press (2022). 10.1017/9781009157926

[4] Health Effects Institute. State of Global Air 2020. Special Report. Boston, MA: Health Effects Institute (HEI) (2020).

[5] Zhao Q Guo Y Ye T Gasparrini A Tong S Overcenco A. Global, regional, and national burden of mortality associated with non-optimal ambient temperatures from 2000 to 2019: a three-stage modelling study. Lancet Planet Heal. (2021) 5:e415–25. 10.1016/S2542-5196(21)00081-4 34245712

[6] Clayton S. Mental health on a changing planet. In: Planetary Health. Island Press (2020). p. 221–44. 10.5822/978-1-61091-967-8_9

[7] Berry HL Waite TD Dear KBG Capon AG Murray V. The case for systems thinking about climate change and mental health. Nat Clim Chang. (2018) 8:282–90. 10.1038/s41558-018-0102-4

[8] IPCC. IPCC Press Conference for CLIMATE CHANGE 2022: Mitigation of Climate Change. (2022). Available online at: https://www.youtube.com/watch?v=STFoSxqFQXU (accessed August 15, 2022).

[9] Vohra K Vodonos A Schwartz J Marais EA Sulprizio MP Mickley LJ. Global mortality from outdoor fine particle pollution generated by fossil fuel combustion: Results from GEOS-Chem. Environ Res. (2021) 195:110754. 10.1016/j.envres.2021.110754 33577774

[10] Erba S Pagliano L. Combining sufficiency, efficiency and flexibility to achieve positive energy districts targets. Energies. (2021) 14:4697. 10.3390/en14154697

[11] Vineis P Romanello M Michelozzi P Martuzzi M. Health co-benefits of climate change action in Italy. Lancet Planet Health. (2022) 6:e293–e294. 10.1016/S2542-5196(22)00061-4 35397213

[12] Vineis P Beagley J Bisceglia L Carra L Cingolani R Forastiere F. Strategy for primary prevention of non-communicable diseases (NCD) and mitigation of climate change in Italy. J Epidemiol Community Health. (2021) 75:917–24. 10.1136/jech-2020-215726 33927002 PMC8372375

[13] IPCC. Climate change 2021: The physical science basis. In: Masson-Delmotte V Zhai P Pirani A Connors SL Pean C Berger S Caud N Chen Y Goldfarb L Gomis MI Huang M Leitzell K Lonnoy E Matthews JBR Maycocl TK Waterfield T Yelekci Yu R Zhou B, editors. Contribution of Working Group I to the Sixth Assessment Report of the Intergovernmental Panel on Climate Change. Cambridge; New York, NY: Cambridge University Press (2022). 10.1017/9781009157896

[14] Castillo MD Anenberg SC Chafe ZA Huxley R Johnson LS Kheirbek I. Quantifying the health benefits of urban climate mitigation actions: current state of the epidemiological evidence and application in health impact assessments. Front Sustain Cities. (2021) 3. 10.3389/frsc.2021.768227

[15] Cromar KR Anenberg SC Balmes JR Fawcett AA Ghazipura M Gohlke JM. Global health impacts for economic models of climate change: a systematic review and meta-analysis. Ann Am Thorac Soc. (2022) 19:1203–12. 10.1513/AnnalsATS.202110-1193OC 35073249 PMC9278641

[16] Gallagher CL Holloway T. Integrating air quality and public health benefits in US decarbonization strategies. Front Public Heal. (2020) 8:563358. 10.3389/fpubh.2020.563358 33330312 PMC7717953

[17] Friel S. Climate Change and the People's Health. New York, NY: Oxford University Press (2019).

[18] Clark WC van Kerkhoff L Lebel L Gallopin GC. Crafting usable knowledge for sustainable development. Proc Natl Acad Sci U S A. (2016) 113:4570–8. 10.1073/pnas.1601266113 27091979 PMC4855559

[19] Bai X Surveyer A Elmqvist T Gatzweiler FW Güneralp B Parnell S. Defining and advancing a systems approach for sustainable cities. Curr Opin Environ Sustain. (2016) 23:69–78. 10.1016/j.cosust.2016.11.010

[20] Nieuwenhuijsen MJ. Urban and transport planning pathways to carbon neutral, liveable and healthy cities; a review of the current evidence. Environ Int. (2020) 140:105661. 10.1016/j.envint.2020.105661 32307209

[21] Howarth C Lane M Morse-Jones S Brooks K Viner D. The ‘co’ in co-production of climate action: challenging boundaries within and between science, policy and practice. Glob Environ Chang. (2022) 72:102445. 10.1016/j.gloenvcha.2021.102445

[22] Daszkiewicz C. Transdisciplinarity, Co-production, Co-exploration: Integrating Knowledge Across Science, Policy Practice in FRACTAL (2019). Available online at: https://www.weadapt.org/knowledge-base/cities-and-climate-change/transdisciplinarity-co-production-and-co-exploration (accessed August 15, 2022).

[23] Vogel JM Smith JB O'Grady M Fleming P Heyn K Adams A. Actionable Science in Practice: Co-Producing Climate Change Information for Water Utility Vulnerability Assessments. Water Utility Climate Alliance (2015). Available online at: http://www.wucaonline.org

[24] de Nazelle A Weinmayr G Roca-Barcel? A Roscoe C Negev M Ebi K. Health Cobenefits of Climate Action Through Co-production and Systems Thinking (2021). Available online at: https://www.iseepi.org/docs/ISEE_ISUH_ClimateChange_Health_PolicyBrief_forStakeholders.pdf (accessed August 12, 2022).

[25] Negev M Zea-Reyes L Caputo L Weinmayr G Potter C de Nazelle A. Barriers and enablers for integrating public health cobenefits in urban climate policy. Annu Rev Public Health. (2022) 43:255–70. 10.1146/annurev-publhealth-052020-010820 34936826

[26] de Nazelle A Roscoe CJ Roca-Barcel? A Sebag G Weinmayr G Dora C. Urban climate policy and action through a health lens—an untapped opportunity. Int J Environ Res Public Health. (2021) 18:12516. 10.3390/ijerph182312516 34886242 PMC8657069

[27] Belesova K Haines A Ranganathan J Seddon J Wilkinson P. Monitoring environmental change and human health: planetary health watch. Lancet. (2020) 395:96–8. 10.1016/S0140-6736(19)33042-9 31929019

[28] Marambe B. Sri Lanka Needs a Return to “Good Agricultural Practices.” Organ Prof Assoc Sri Lanka (2022). Available online at: https://www.researchgate.net/publication/360127202 (accessed August 15, 2022).

[29] Stoknes P. What We Think About When We Try Not To Think About Global Warming: Toward a New Psychology of Climate Action. Chelsea Green Publishers (2015). Available online at: https://www.chelseagreen.com/product/what-we-think-about-when-we-try-not-to-think-about-global-warming/

[30] Kopatz M. Ökoroutine - Damit wir tun, was wir für richtig halten. Oekom Verlag (2016).

[31] Milkoreit M Hodbod J Baggio J Benessaiah K Calderón-Contreras R Donges JF. Defining tipping points for social-ecological systems scholarship—an interdisciplinary literature review. Environ Res Lett. (2018) 13:033005. 10.1088/1748-9326/aaaa75

[32] Thaler RH Sunstein CR. Nudge: Improving Decisions About Health, Wealth, and Happiness. Penguin Books (2009).

